# mPulse Mobile Sensing Model for Passive Detection of Impulsive Behavior: Exploratory Prediction Study

**DOI:** 10.2196/25019

**Published:** 2021-01-27

**Authors:** Hongyi Wen, Michael Sobolev, Rachel Vitale, James Kizer, J P Pollak, Frederick Muench, Deborah Estrin

**Affiliations:** 1 Cornell Tech Cornell University New York, NY United States; 2 Feinstein Institutes for Medical Research Northwell Health Manhasset, NY United States; 3 The Partnership to End Addiction New York, NY United States

**Keywords:** mobile sensing, digital phenotyping, impulse control, impulsivity, self-regulation, self-control, mobile health, mHealth

## Abstract

**Background:**

Mobile health technology has demonstrated the ability of smartphone apps and sensors to collect data pertaining to patient activity, behavior, and cognition. It also offers the opportunity to understand how everyday passive mobile metrics such as battery life and screen time relate to mental health outcomes through continuous sensing. Impulsivity is an underlying factor in numerous physical and mental health problems. However, few studies have been designed to help us understand how mobile sensors and self-report data can improve our understanding of impulsive behavior.

**Objective:**

The objective of this study was to explore the feasibility of using mobile sensor data to detect and monitor self-reported state impulsivity and impulsive behavior passively via a cross-platform mobile sensing application.

**Methods:**

We enrolled 26 participants who were part of a larger study of impulsivity to take part in a real-world, continuous mobile sensing study over 21 days on both Apple operating system (iOS) and Android platforms. The mobile sensing system (mPulse) collected data from call logs, battery charging, and screen checking. To validate the model, we used mobile sensing features to predict common self-reported impulsivity traits, objective mobile behavioral and cognitive measures, and ecological momentary assessment (EMA) of state impulsivity and constructs related to impulsive behavior (ie, risk-taking, attention, and affect).

**Results:**

Overall, the findings suggested that passive measures of mobile phone use such as call logs, battery charging, and screen checking can predict different facets of trait and state impulsivity and impulsive behavior. For impulsivity traits, the models significantly explained variance in sensation seeking, planning, and lack of perseverance traits but failed to explain motor, urgency, lack of premeditation, and attention traits. Passive sensing features from call logs, battery charging, and screen checking were particularly useful in explaining and predicting trait-based sensation seeking. On a daily level, the model successfully predicted objective behavioral measures such as present bias in delay discounting tasks, commission and omission errors in a cognitive attention task, and total gains in a risk-taking task. Our models also predicted daily EMA questions on positivity, stress, productivity, healthiness, and emotion and affect. Perhaps most intriguingly, the model failed to predict daily EMA designed to measure previous-day impulsivity using face-valid questions.

**Conclusions:**

The study demonstrated the potential for developing trait and state impulsivity phenotypes and detecting impulsive behavior from everyday mobile phone sensors. Limitations of the current research and suggestions for building more precise passive sensing models are discussed.

**Trial Registration:**

ClinicalTrials.gov NCT03006653; https://clinicaltrials.gov/ct2/show/NCT03006653

## Introduction

Mobile health (mHealth) technology has demonstrated the ability of smartphone apps and sensors to collect high-fidelity and high-frequency data pertaining to patient activity, behavior, symptoms, cognition, and context [1]. Mobile sensing, in particular, has the ability to collect data objectively and continuously during the lived experience of individuals. In behavioral and mental health, digital phenotyping [2-4] or personal sensing [5] has been proposed as an approach to quantify the “moment-by-moment and continuous individual-level human phenotype” using data from sensors on smartphones. Building on this potential, prior research using mobile sensing technology focused on specific psychological disorders [6-11] or general mental and physical well-being [12-14].

One construct that has not been rigorously examined is impulsivity and impulsive behavior. Impulsivity is a multidimensional construct primarily characterized by the inability to inhibit acting on short-term temptations despite long-term consequences or loss of potential gains. Consequently, it is the hallmark feature of self-regulation failures that lead to poor health decisions and outcomes, making understanding and treating impulsivity one of the most important constructs to tackle in building a culture of health [15-18]. Across studies and subtypes, highly impulsive individuals are significantly more likely to suffer from obesity, type II diabetes, substance use disorder, attention-deficit/hyperactivity disorder, gambling problems, bipolar disorder, borderline personality disorder, and suicidal behaviors, among others [17,19-21]. Prediction of impulsive behavior is nevertheless challenging due to the multidimensional and heterogeneous nature of the impulsivity construct and different manifestations of state impulsivity [20,22]. Such impulsive behavior includes the traits of urgency, lack of planning or premeditation, lack of perseverance, inattention, present and future discounting, response inhibition, and sensation seeking. Passive detection of impulsive behavior is a crucially important research goal given the widespread negative consequences of impulsivity.

Potential behavioral biomarkers of impulsive behavior are intuitively present in most interactions with digital technology. Mobile sensing may be especially useful for assessing impulsive behavior indicative of digital addiction, such as loss of control over mobile phone use, interference with other activities, and repeated phone checking. Objectively quantifying phone usage can further help inform the debate on the existence of digital addiction [23] and identify distinct problematic uses of smartphones. Preliminary evidence suggested a link between impulsivity traits and use of mobile devices. Studies of self-reported phone usage conducted by Billieux et al [24,25] revealed a direct relationship between the inability to delay gratification and different patterns of mobile phone use. In other studies, mobile analytics features, such as latency to respond to a text, were shown to predict personality traits associated with impulsivity, such as extraversion and neuroticism [26-29].

We developed a mobile sensing system—mPulse—to remotely monitor impulsivity on both Apple operating system (iOS) and Android platforms. Our system was designed based on data that are pervasive and available across both iOS and Android platforms and can be used to measure signals of daily activities, social interactions, and digital addiction. We selected call logs, battery charging, and screen checking as the mobile sensor data sources. We conducted a 3-week exploratory study with 26 participants as part of a larger mHealth study of impulsive behavior called the Digital Marshmallow Test (DMT) [30]. To validate the mobile sensing model, we used mobile sensing features to predict common self-reported impulsivity traits, objective behavioral and cognitive measures, and ecological momentary assessment (EMA) of impulsivity and constructs related to impulsive behavior (ie, risk-taking, attention, and affect).

## Methods

### Background

The DMT study by Sobolev et al [30] was designed to develop and test remote assessment of impulsivity using both iOS and Android applications for widespread dissemination to researchers, clinicians, and the general public. The DMT study included a baseline laboratory assessment and a 21-day study using the DMT mobile app [30,31]. Additional details can be found in the paper describing validation of the DMT app [30] and on the Open Science Framework [31].

### Participants

Of the 116 participants enrolled in the DMT study, a subsample of 26 participants enrolled in this passive sensing study. The subsample included 14 females, 10 males, and 2 participants who refused to disclose, and the average age of the participants was 39.1 (SD 14.16) years. Twenty-two participants owned Apple (iOS) phones (ie, iPhones) and 4 owned Android phones. We compared the baseline subjective trait assessments of trait impulsivity and impulsive behavior between the current subsample of participants and the full sample and found no significant differences between the groups.

### Data Sources

The DMT study included three main data sources, which we used as dependent variables in this study: (1) subjective, self-reported trait impulsivity assessments performed at baseline in the lab; (2) behavioral and cognitive active tasks performed daily on the DMT mobile app; and (3) self-reports, ecological momentary assessments (EMAs), and the Photographic Affect Meter (PAM) performed daily on the DMT mobile app.

#### Subjective, Self-Reported Trait Measures (Lab)

The DMT study included the two most popular self-report generalized impulsivity trait assessments collected in a lab setting: the 15-item short form of the Barratt Impulsiveness Scale (BIS-15) and the UPPS.

The BIS-15 [32] measures three aspects of impulsivity: attention (inability to focus attention or concentrate), motor (acting without thinking), and nonplanning (lack of future orientation or forethought).

The UPPS impulsive behavior scale [33] assesses impulsivity on subscales pertaining to urgency (acting rashly under conditions of negative affect), lack of premeditation (difficulty in thinking and reflecting on consequences of an act), lack of perseverance (inability to remain focused on a task), and sensation seeking (tendency and openness to try and enjoy exciting or dangerous activities).

#### Behavioral and Cognitive Active Tasks (DMT App)

The DMT app included an adaptation of three exploratory, lab-based behavioral and cognitive measures related to impulse control to mobile devices, called “active tasks”: (1) a mobile Balloon Analogue Risk Task (mBART [34]), (2) a mobile go/no-go (mGNG [35]) task, and (3) a mobile delay discounting (mDD [36]) task. The mobile versions are exploratory and were partially validated as part of the DMT study (see the DMT study [30] for more details on each of these measures).

The mBART measures how individuals balance the potential for reward and loss via a simulated test where the participant can earn virtual money by pumping a balloon. It is based on the BART [34]. The mBART includes 15 trials and lasts approximately 2 minutes. We recorded the number of pumps, which indicates risk taking, and the total gains in the task for each trial.

The mGNG is a measure of attention and response control. It is based on the GNG task [35]. The mGNG included 75 trials, each of which had the following sequence: fixation cross (250 ms), blank screen (250 ms), vertical or horizontal cue (white rectangle) for 1 of 6 stimulus-onset asynchronies (100 ms, 200 ms, 300 ms, 400 ms, 500 ms, and 750 ms), go or no-go target (green or blue rectangle, respectively) until participant responds or 500 ms, and an intertrial interval (250 ms). Participants were instructed to respond by pressing the screen as fast as possible to green, but not to blue, targets. Cues signal a target at 70% probability (horizontal: go; vertical: no-go). We recorded the commission and omission errors and response latency before they reacted to the targets.

The mDD task is used to measure the ability to delay immediate, smaller, and shorter monetary and time-based rewards for longer, time-lapsed, but larger rewards. It is based on DD tasks that were used in research on addiction [36]. We used the algorithm as described by Frye and colleagues [37]. In the mDD task, participants were given five choices between a smaller, hypothetical monetary or time-based reward that varied from trial to trial based on the previous response and a larger, fixed reward that remained the same throughout all of the trials. We recorded the propensity of choosing an immediate, smaller reward in each trial.

#### Self-Report, EMA, and PAM (DMT App)

The DMT app included self-reports, EMAs, and PAM.

EMAs were based on a semantic differential scale and questions consisted of two opposite feelings, thoughts, or behaviors [38]. We measured five items from 0 (most positive) to 10 (most negative): (1) focused–distracted, (2) intentional–impulsive, (3) cautious–thrill-seeking, (4) engaged–bored, and (5) determined–aimless. These items were measured twice daily with respect to the feeling in the present moment in the morning (AM) and evening (PM).

Self-reported questions were also based on a semantic differential scale [38]. We measured five items from 0 (most positive) to 10 (most negative): (1) positive–negative, (2) intentional–impulsive, (3) productive–unproductive, (4) relaxed–stressed, and (5) healthy–unhealthy. These items were self-reported based on the general feeling in the previous day.

PAM was designed for momentary response where users choose an image that best represents their emotion at a given time [39]. We used the positive and negative affect scores from PAM that have been validated to correspond to the short version of the Positive and Negative Affect Schedule (PANAS) [40].

#### Descriptive Statistics of DMT Data

We analyzed the correlations between different self-reports (BIS-15 and UPPS) and behavioral measures (BAR and GNG) in the full sample of the DMT study (N=116) because it provides better estimates than the subsample of 26 participants in this study. Overall, our results corresponded to previous research on impulsivity by demonstrating high correlations between different self-reports but low correlations between behavioral measures and self-reports [22]. A full description of these results can be found in the paper describing the DMT study [30].

### mPulse Sensing System and Data

#### AWARE Framework

AWARE Framework is an open-source framework used to develop an extensible and reusable platform for capturing context on mobile devices [41]. It is available on both iOS and Android platforms as an installable app that collects phone sensor data (eg, activity and screen checking). In this study, we used the AWARE app to record call logs, battery charging, and screen checking locally on participants’ phones.

#### Sensor Data

**Figure 1:**
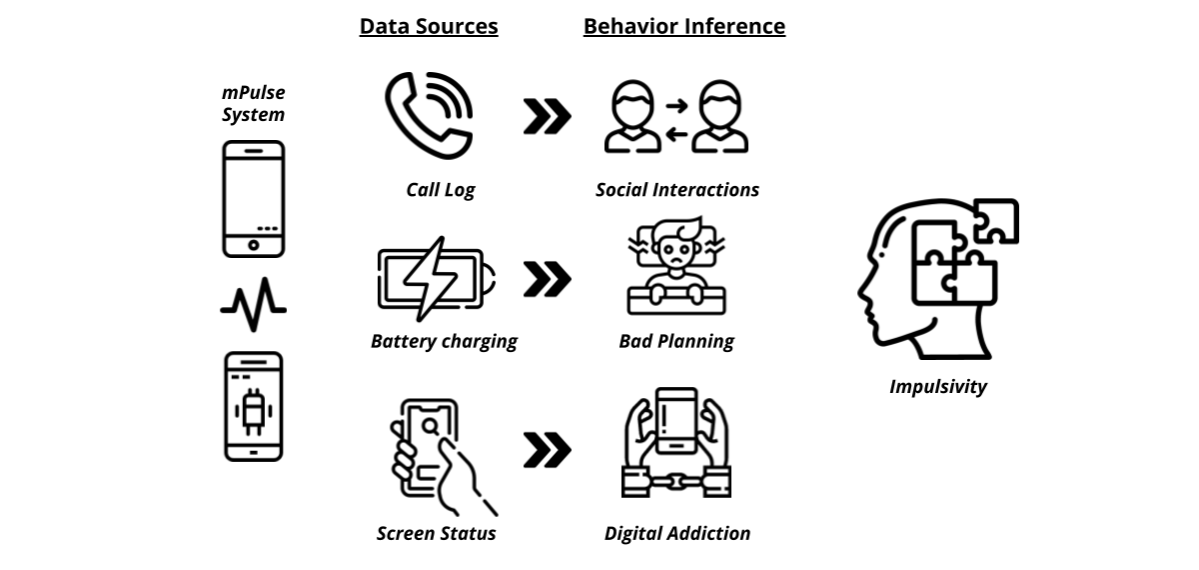
Conceptual framework of passive sensor data and inferred behavior.

Our goal was to create sensing models that can effectively transform raw sensor data collected from mobile phones into measurable outcomes of clinical interest. We focused on data that are pervasively available across both iOS and Android platforms while minimizing battery consumption beyond the normal use of mobile devices and protecting user privacy. Therefore, despite the relevance of data sources such as accelerometers and location data for physical activity, mobility, and motor impulsivity, we elected not to include these data sources in the passive sensing model in this study. Eventually, three types of sensor data were identified and implemented in the mPulse system (Figure 1) for these purposes: call logs, battery charging, and screen checking.

##### Call Logs

Call logs are indicators of social interactions [42] and are frequently used in mobile sensing studies. Prior research, for example, identified negative correlations between frequency of incoming and outgoing calls and depressive symptoms in both clinical [43] and nonclinical [44] samples. In the mPulse system, we recorded time stamps of each call the participants sent or received and their durations. Any identifiable information, such as phone number or contact’s name, were not recorded by the passive app.

##### Battery Charging

Battery logs are an indicator of daily activities [42]. We identified battery management as a potential indicator of self-regulation in the context of phone usage and planning. In the system, we recorded the time stamps and durations of battery charging events. We observed several instances of a charging event with a duration of 1 second followed by a longer charging event, which we suspected were caused by system error. Thus, we removed charging events that were shorter than 10 seconds. Using these criteria, 16.5% of the raw data were filtered out.

##### Screen Checking

Screen checking can serve as an indicator of digital and mobile addiction. For example, a previous study demonstrated that individuals with smartphone addiction presented with some symptoms common to substance- and addictive-related disorders such as compulsive behavior, tolerance, and withdrawal [45]. In the mPulse system, we measured screen checking by collecting the number of screen unlocks and the duration of each unlock session. Notification-induced screen-on events were intentionally excluded. We removed screen unlock sessions longer than 2 hours, which are triggered by unrelated usage, such as continuous use of the phone for navigation while driving. This resulted in the removal of only 0.4% of the data.

#### Feature Extraction

**Figure 2:**
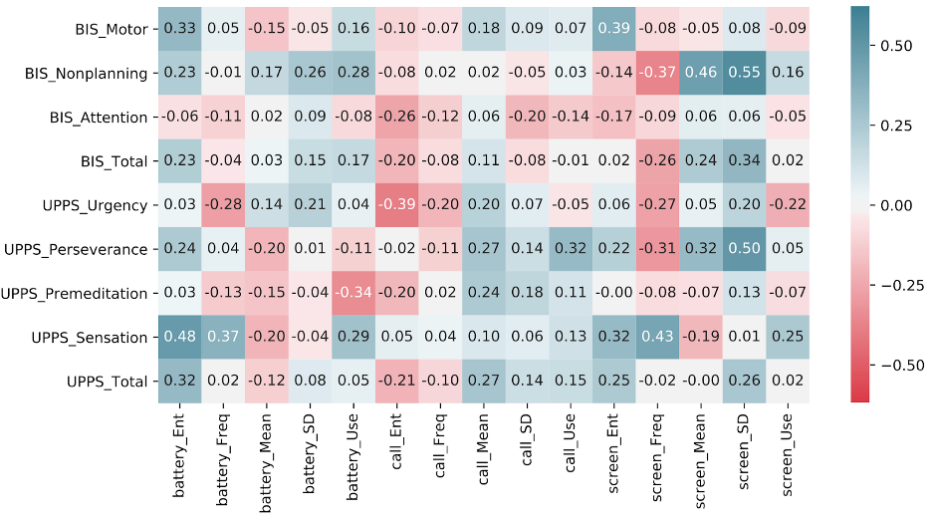
Correlation between the 15 features of mobile sensor data and trait impulsivity scales (15-item short form of the Barratt Impulsiveness Scale [BIS-15] and UPPS) and subscales. Ent: entropy; Freq: frequency per hour; Mean: use mean; SD: use deviations; Use: use duration per hour.

From the passive data, we extracted the same set of features for all sensor data, namely usage, frequency, entropy, mean, and standard deviation. This resulted in 15 passive features for the analysis:

- Use duration and frequency per hour: normalized duration and frequency for each hour—that is, the summation of sensor event durations and occurrences divided by total hours of data collected from each individual, respectively. For example, screen unlocks use duration per hour (denoted as screen_Use in Figure 2) refers to the average amount of time the screen was unlocked in each hour; battery_Freq refers to the number of battery charges triggered by a user in each hour.
- Use mean and standard deviation: used to measure individual usage baselines and variances. We calculated the means and standard deviations of the event durations (unit in hours) across the study for each participant. For example, screen_Mean=0.1 means that the average screen unlock duration was 0.1×60=6 minutes.
- Entropy: calculated from the possibility distribution of event occurrences over 24 hours. The intuition is that if the occurrences of the events distribute more uniformly across the day, the pattern is more random (higher entropy); otherwise, if the events occur more frequently at certain hours of the day, the pattern is more controlled (lower entropy). This was inspired by the use of the entropy feature in prior mobile sensing research to measure variability of time the participant spent at the location clusters [8].

#### Descriptive Statistics of Mobile Sensing

Means and standard deviations across individuals for the mobile sensing features are presented in Table 1. To predict assessment of trait impulsivity and impulsive behavior (BIS-15 and UPPS), we used averages across individuals as predictor variables. For predicting daily features, such as active tasks and EMA questions, we used the 24-hour window before the morning assessment.

**Table 1:** Descriptive statistics of mobile sensor data and features.

Descriptive statistics	Battery charging, mean (SD)	Call logs, mean (SD)	Screen checking, mean (SD)
Usage (per hour)	0.30 (0.12)	0.02 (0.01)	0.17 (0.08)
Frequency (number per hour)	0.20 (0.15)	0.38 (0.28)	1.97 (1.22)
Mean (duration per activity in hours)	2.02 (1.33)	0.06 (0.04)	0.11 (0.05)
Deviations (duration per activity in hours)	2.81 (1.21)	0.15 (0.18)	0.17 (0.07)
Entropy	2.65 (0.24)	2.52 (0.25)	2.89 (0.10)

## Results

### Predicting Clinical Assessments of Impulsivity Trait

In this section, we evaluate the value of mobile sensing in explaining and predicting trait impulsivity. We first examined the correlations between mobile sensing features and different components of trait impulsivity. Next, we compared the goodness of fit for regression models using mobile sensing features as predictors. Finally, we validated the predictive power of such models using leave-one-subject-out (LOSO) cross-validation.

#### Correlations Analysis

We found significant correlations between passive data and five of the components of trait impulsivity: (1) motor positively correlated with the entropy features extracted from screen checking (*r*=0.39, *P*=.05), suggesting that the temporal distribution of phone usage was associated with the trait of acting without thinking; (2) nonplanning correlated with several passive features, including the usage mean (*r*=0.46, *P*=.02) and usage deviations (*r*=0.55, *P*=.004) of screen-checking duration; (3) sensation seeking positively correlated with battery charging entropy (*r*=0.48, *P*=.01) and the screen-checking frequency (*r*=0.43, *P*=.03); (4) urgency negatively correlated with call entropy (*r*=–0.39, *P*=.04); and (5) perseverance positively correlated with the standard deviation of screen checking (*r*=0.50, *P*=.01). The full correlation table is shown as Figure 2.

#### Regression Analysis

We performed a multivariate regression analysis to examine the power of extracted mobile sensing features from day-to-day phone usage to explain components of trait impulsivity. Features were standardized across samples. Given our small sample size, we first used Lasso regularization to prevent overfitting by selecting the most important features. The same penalty threshold was used across all models (α=.05). We then used a linear regression model with ordinary least squares to estimate the trait impulsivity scores from the selected features. Model performance was evaluated against adjusted *R*^2^ and is summarized in Table 2.

Our analysis discovered four significant models: (1) sensation seeking (*F*_9,16_=5.54; *P*=.002), with screen-checking frequency (β=.39; *P*=.01), call entropy (β=−.60; *P*=.001), and battery usage (β=.27; *P*=.01) as significant predictors; (2) perseverance (*F*_4,21_=3.35; *P*=.03), with deviation of screen-checking duration as a significant predictor (β=.22; *P*=.006); (3) motor (*F*_6,19_=2.42, *P*=.07), with screen entropy as a significant predictor (β=.24; *P*=.047); and (4) planning (*F*_4,21_=3.76; *P*=.02), with deviation of screen-checking duration as a significant predictor (β=.33; *P*=.002).

**Table 2:** Descriptive statistics of laboratory subjective impulsivity and impulsive behavior trait, and regression analysis of mobile sensor data as predictors of impulsivity trait scales and subscales.

Scale and subscale		Descriptive statistics, mean (SD)	Regression summary	Significant features
**BIS-15**		1.77 (0.36)	*F*_4,21_=1.36; *P*=.28; *R*^2^=0.055	None
	Motor	1.74 (0.45)	*F*_6,19_=2.42; *P*=.07; *R*^2^=0.254	Screen entropy (β=.24; *P*=.05)
	Nonplanning	1.84 (0.54)	*F*_4,21_=3.76; *P*=.02; *R*^2^=0.307	Screen deviations (β=.33; *P*=.002)
	Attention	1.66 (0.49)	*F*_2,23_=1.19; *P*=.32; *R*^2^=0.015	None
**UPPS**		2.04 (0.36)	*F*_4,21_=3.48; *P*=.02; *R*^2^=0.284	Call entropy (β=−.21; *P*=.01)
	Urgency	2.07 (0.66)	*F*_7,18_=1.16; *P*=.21; *R*^2^=0.135	Call entropy (β=−.39; *P*=.04)
	Lack of perseverance	1.57 (0.42)	*F*_4,21_=3.35; *P*=.03; *R*^2^=0.273	Screen deviations (β=.22; *P*=.006)
	Lack of premeditation	1.73 (0.35)	*F*_4,21_=1.27; *P*=.31; *R*^2^=0.042	None
	Sensation seeking	2.66 (0.64)	*F*_9,16_=5.54; *P*=.002; *R*^2^=0.621	Battery frequency (β=.27; *P*=.01); screen usage (β=.39; *P*=.01); call entropy (β=−.60; *P*=.001)

#### Prediction Analysis

LOSO cross-validation was performed to further examine the predictive power of the passive sensing features for out-of-sample data. We trained a separate linear support vector regression model for each set of passive features for 25 participants and tested it on the 1 remaining participant. We ran the same procedure 26 times to obtain predicted scores for all 26 participants. Model performance was evaluated against mean absolute error (MAE) and Pearson *r*. We found that the passive model predicted only the sensation-seeking trait with a MAE of 0.479. The correlation between predicted scores and true scores was significant (*r*=0.425; *P*=.03).

### Predicting Daily Measures of State Impulsivity

#### Descriptive Statistics on Daily Variables

Descriptive statistics on daily variables used for prediction of state impulsivity are presented in Table 3.

**Table 3:** List of features from ecological momentary assessments and active tasks.

Features		Description	Descriptive statistics, mean (SD)
**Present moment semantic differentials^a^**			
	Focused–distracted	Present moment distracted score	AM: 3.23 (2.45); PM: 3.71 (2.74)
	Intentional–impulsive	Present moment impulsive score	AM: 3.86 (2.75); PM: 4.47 (2.93)
	Cautious–thrill-seeking	Present moment thrill-seeking score	AM: 3.63 (2.20); PM: 3.63 (3.68)
	Engaged–bored	Present moment bored score	AM: 3.24 (2.11); PM: 3.23 (2.33)
	Determined–aimless	Present moment aimless score	AM: 2.74 (2.04); PM: 3.08 (2.19)
**Previous day semantic differentials^a^**			
	Positive–negative	Previous day negativity score	2.59 (2.11)
	Intentional–impulsive	Previous day impulsive score	3.95 (2.92)
	Productive–unproductive	Previous day unproductive score	2.47 (1.99)
	Relaxed–stressed	Previous day stressed score	4.64 (2.84)
	Healthy–unhealthy	Previous day unhealthy score	3.92 (2.50)
**PAM^b^**			
	Positive affect	Positive affect score from PAM	9.25 (3.50)
	Negative affect	Negative affect score from PAM	5.79 (3.66)
**mBART^c^**			
	Risk-taking	Average number of pumps across all trials	3.89 (1.09)
	Total gains	Average total gain across all trials	10.31 (2.73)
**mGNG^d^**			
	Response latency	Average response time across all trials	423.99 ms (67.70)
	Commission error	Proportion of “go” errors across all “go” trials	0.02 (0.06)
	Omission error	Proportion of “no-go” errors across all “no-go” trials	0.02 (0.03)
**mDD^e^**			
	Present bias	Average propensity to choose immediate reward across all trials	0.34 (0.18)

^a^Measured on a scale from 0-10, with 0=most positive and 10=most negative.

^b^PAM: Photographic Affect Meter.

^c^mBART: mobile Balloon Analogue Risk Task.

^d^mGNG: mobile go/no-go task.

^e^mDD: mobile delay discounting task.

#### Predicting EMA

We used a generalized estimating equation (GEE) model to take into account the intraclass correlations for individual differences. We performed a multivariate regression analysis for five daily semantic differentials and positive and negative affect measures. We further performed a binary classification task by labeling samples with 1=higher than the median value and 0=lower than the median value for each daily measure. We used a logistic regression model and LOSO cross-validation. The full results are reported in Table 4.

Our analysis discovered three significant models for morning and evening semantic differentials: (1) focused–distracted (AM: *r*=0.276, *P*<.001, 83% accuracy; PM: *r*=0.194, *P*=.002, 74% accuracy); (2) cautious–thrill-seeking (AM: *r*=0.245, *P*<.001, 86% accuracy; PM: *r*=0.361, *P*<.001, 87% accuracy); and (3) determined–aimless (AM: *r*=0.360, *P*<.001, 94% accuracy; PM: *r*=0.217, *P*<.001, 91% accuracy). Our analysis also discovered four significant models for previous day semantic differentials: (1) positive–negative (*r*=0.316, *P*<.001, 84% accuracy); (2) relaxed–stressed (*r*=0.377, *P*<.001, 63% accuracy); (3) healthy–unhealthy (*r*=0.248, *P*<.001, 76% accuracy); and (4) productive–unproductive (*r*=0.271, *P*<.001, 92% accuracy). Models for positive affect (*r*=0.143, *P*<.001, 72% accuracy) and negative affect (*r*=0.171, *P*<.001, 72% accuracy) were also significant with similar effects. Notably, the models were not significant for predicting intentional–impulsive (*r*=0.057, *P*=.34, 68% accuracy).

**Table 4:** Regression analysis and classification of mobile sensor data as predictors of daily ecological momentary assessment questions for semantics differentials and the Photographic Affect Meter (PAM).

Features		Generalized estimating equation regression summary (Pearson *r*, within-group correlation)	Classification accuracy (SD) across individuals
**Present moment semantic differentials (AM/PM)**			
	Focused–distracted	AM: *r*=0.276, *P*<.001, 0.388; PM: *r*=0.194, *P*=.002, 0.388	AM: 0.83 (0.21); PM: 0.74 (0.26)
	Intentional–impulsive	AM: *r*=–0.04, *P*=.50, 0.743; PM: *r*=0.04, *P*=.51, 0.753	AM: 0.80 (0.28); PM: 0.64 (0.29)
	Cautious–thrill-seeking	AM: *r*=0.245, *P*=<.001, 0.633; PM: *r*=0.361, *P*=<.001, 0.631	AM: 0.86 (0.17); PM: 0.87 (0.16)
	Engaged–bored	AM: *r*=0.273, *P*=<.001, 0.329; PM: *r*=0.061, *P*=.322, 0.481	AM: 0.86 (0.14); PM: 0.84 (0.18)
	Determined–aimless	AM: *r*=0.360, *P*<.001, 0.185; PM: *r*=0.217, *P*=<.001, 0.285	AM: 0.94 (0.12); PM: 0.91 (0.15)
**Previous day semantic differentials**			
	Positive–negative	*r*=0.316, *P*<.001, 0.157	0.84 (0.17)
	Intentional–impulsive	*r*=0.057, *P*=.34, 0.794	0.68 (0.28)
	Productive–unproductive	*r*=0.271, *P*<.001, 0.161	0.92 (0.10)
	Relaxed–stressed	*r*=0.377, *P*<.001, 0.134	0.63 (0.22)
	Healthy–unhealthy	*r*=0.248, *P*<.001, 0.242	0.76 (0.21)
**PAM**			
	Positive affect	*r*=0.143, *P*<.001, 0.112	0.72 (0.15)
	Negative affect	*r*=0.171, *P*<.001, 0.114	0.72 (0.15)

### Predicting Daily Active Tasks

We used a GEE model to take into account the intraclass correlations for individual differences. We performed an exploratory multivariate regression analysis for six features from the three behavioral and cognitive active tasks: mBART, mGNG, and mDD. We further performed a binary classification task by labeling samples with 1=higher than the median value and 0=lower than the median value for each daily measure. We used a logistic regression model and LOSO cross-validation. The full results are reported in Table 5.

Our analysis discovered five significant models that varied greatly in classification accuracy: (1) total gains from mBART (*r*=0.326, *P*<.001, 59% accuracy); (2) response latency (*r*=0.334, *P*<.001, 58% accuracy), commission error (*r*=0.155, *P*=.07, 89% accuracy), and omission error (*r*=0.361, *P*<.001, 87% accuracy) from mGNG; and (3) present bias from mDD (*r*=0.792, *P*<.001, 84% accuracy). Risk-taking from mBART was not statistically significant (*r*=0.067, *P*=.43, 48% accuracy).

**Table 5:** Regression analysis and classification of mobile sensor data as predictors of daily active behavioral and cognitive tasks.

Active tasks		Generalized estimating equation regression summary (Pearson *r*, within-group correlation)	Classification accuracy (SD) across individuals
**mBART^a^**			
	Risk-taking	*r*=0.067, *P*=.43, 0.762	0.48 (0.23)
	Total gains	*r*=0.326, *P*<.001, 0.505	0.59 (0.27)
**mGNG^b^**			
	Response latency	*r*=0.334, *P*<.001, 0.765	0.58 (0.31)
	Commision error	*r*=0.155, *P*=.07, 0.415	0.89 (0.16)
	Omission error	*r*=0.361, *P*<.001, 0.121	0.87 (0.13)
**mDD^c^**			
	Present bias	*r*=0.792, *P*<.001, –0.051	0.84 (0.33)

^a^mBART: mobile Balloon Analogue Risk Task.

^b^mGNG: mobile go/no-go task.

^c^mDD: mobile delay discounting task.

## Discussion

This exploratory study examined the potential of detecting and monitoring state impulsivity and impulsive behavior in daily life using continuous and ubiquitous mobile sensing. We explored the predictive power of the mobile sensing system and model we developed (mPulse). We discovered relationships between passive mobile sensor data and self-reported impulsivity traits, EMA of impulsive behavior, and mobile behavioral and cognitive active tasks of risk-taking, attention, and time preference.

### Principal Results

This is the first study to examine the relationship between passive mobile phone data, daily self-reports and self-report measures of trait impulsivity, and exploratory, objective, active mobile measures of impulsivity. Overall, our findings suggest that passive measures of mobile phone use such as call logs, battery usage, and screen on-off metrics can predict different facets of impulsivity and impulsive behavior in nonclinical samples. This study adds to the emerging literature on mobile phone phenotyping using ubiquitous sensor data as well as to the measurement of impulsive behavior in daily life [46-48]. Our results can further inform the development of digital interventions for individuals [49-51] by identifying and intervening with potential problematic behavioral patterns before they result in consequences.

First, we investigated the relationship between mobile sensing features and impulsivity traits on the individual level. Our regression models significantly explained variance in sensation-seeking, nonplanning, and lack of perseverance traits, but failed to explain motor, urgency, lack of premeditation, and attention traits. Passive sensing features from call logs, battery charging, and screen checking were particularly useful in explaining and predicting the sensation-seeking trait. The regression model indicated that overall battery charging frequency and screen-checking usage were significant positive predictors of sensation seeking, while call entropy was a significant negative predictor. Cross-validation further confirmed the validity of these mobile sensing features for predicting sensation seeking.

Sensation seeking in itself has multiple facets from thrill-seeking to boredom proneness to disinhibition. Therefore, due to the rewarding nature of interacting with mobile devices, one would expect to discover digital biomarkers of sensation seeking in mobile sensor data. Our results suggest that individuals high in sensation and thrill-seeking may be more prone to repeated phone checking and more intense interactions with their devices when they are using them (eg, less entropy). Previous studies have yielded mixed findings on the relationship between sensation seeking and psychopathology. For example, in a meta-analysis of the UPPS subscales, sensation seeking demonstrated the strongest associations with alcohol and substance use but an overall lower relationship with other clinical conditions than other UPPS traits [51]. It could be that these relationships represent not only maladaptive behaviors but also a desire to seek information, be conscientious at work or with family requests, and stay connected to others. Future studies should collect more information on the interaction between sensation and thrill-seeking and reasons for phone checking to parse out the positive and negative relationships between these passive metrics and outcomes.

Second, we explored the use of mobile sensing features to discover measures that assess state impulsivity and impulsive behavior in daily life. Our mobile sensing model successfully predicted objective behavioral measures, such as present bias in a delay discounting task, commission and omission errors in a cognitive attention task, and total gains in a risk-taking task. Our models also successfully predicted daily EMA questions on positivity, stress, health, and affect. Perhaps most intriguingly, our model failed to predict daily EMA questions designed to measure previous day and present moment impulsivity directly.

This finding indicates that it might be easier to predict constructs related to trait impulse control than self-reported state impulsivity itself in our sample. While studies have revealed that trait impulsivity is highly related to state impulsivity [47,48], there may be more powerful constructs that mediate the relationship between sensors and state impulsivity. For example, studies have revealed a close relationship between affect and impulsive behavior and, separately, between affect and phone sensor data [44], which may have more robust relationships than with state intentionality–impulsivity. It is also possible that because our sample skewed toward intentional versus impulsive responses, we were less able to detect differences. Despite this surprising finding, the data does suggest that combined mobile phone use features are associated with a range of important factors related to well-being, such as perceived productivity. This further highlights the need to personalize passive detection models of state impulsivity or impulsive behavior for the appropriate context, such as substance misuse, productivity, and gambling. It also suggests the need to compare this sample against clinical populations with potentially higher impulsivity scores. Taken together, the exploratory analysis between the passive mobile phone features and daily measures of impulsive behavior revealed that the range of combined mobile phone sensors can predict certain behaviors but that identifying the individual predictors of these components is more challenging.

### Digital Addiction and Problematic Phone Usage

Passive mobile sensing can be particularly useful for detecting signs of digital addiction and problematic phone usage. Digital addiction and excessive phone usage are considered other negative consequences of impulsivity and self-regulation failures [24]. We considered this emerging theoretical relationship in the design of the mPulse sensing model, which provides ecologically valid features such as battery usage and screen checking. Our preliminary results confirmed this hypothesized relationship through the sensation-seeking trait, which can explain reward-based phone usage. The relationship between sensation seeking and screen checking was further evidenced by the significant associations between screen frequency and thrill-seeking EMA. It is also possible to use mobile sensing models to predict consequences of digital addiction, such as daily productivity. There is an opportunity to use our passive sensing models to contribute to the debate on the existence and measurement of digital addiction and distinguish between actual and problematic phone usage [23]. Mobile sensing can help objectively detect signals of problematic phone usage and provide input into personalized interventions to reduce this impulsive behavior [52]. Future research should model and evaluate mobile sensing features as they relate to digital addiction and problematic use of smartphones.

### Challenges of Detecting and Predicting Impulsive Behavior in Daily Life

Our inability to predict traits such as attention and urgency, which should theoretically correlate with mobile sensing features, indicates the challenge of predicting impulsivity using the sensors chosen for the current study. Similarly, our models struggled the most with predicting the EMA question that directly asked participants to self-report the general state impulsivity in the present moment and in the previous day. We suspect this finding might be due to the multidimensional nature of impulsivity and the complex interaction between trait and state impulsivity [20]. While studies showed promising results for measuring momentary impulsivity [46-48], the overall convergence between behavioral and self-report measures of the impulsivity construct remains low [22]. Future research should ideally include larger samples of clinical and nonclinical populations and different measures to discover and model these interactions. Mobile sensing and phenotyping can provide an additional objective method of assessing impulsive behavior. This method can provide further insight into a range of new, unexplored opportunities to understand human behavior and explain impulsive behavior.

### Cross-Platform mHealth and Sensing

One of the primary goals of this study was to design a mobile sensing system and model, supporting both iOS and Android platforms. The majority of foundational research on mobile sensing was examined on a single platform, which limits the generalizability and real-life applicability of the findings. Cross-platform research services more diverse populations and offers different opportunities for passive and active assessment. Given differences between the two operating systems, compromises are required when considering passive sensor data sources to only collect the subset of sensor data that are available on all devices. Android devices in particular offer a wider range of passive sensing modalities, such as app usage and keyboard typing, compared with iOS devices. The mobile sensing capabilities of different platforms, however, continue to evolve and new restrictions might limit future research and replicability of our findings. Passive sensing can only be useful if the environments used to collect the data do not cause the user more burden than other methods of data collection.

### Privacy and Ethical Concerns in Mobile Sensing

More comprehensive sensing suggests greater privacy concerns, as more data related to a person’s life and behavior can be quantified, transmitted, and stored. The intention of collecting passive sensing active behavioral tasks and EMA data was to build and validate digital biomarkers that can assess impulsivity for future intervention and management, and the preliminary results show the promise of such data. Yet, there exist very real possibilities for such data to be used to exploit a user, for example through stimulated impulsive purchasing [53,54] or targeted advertising. These passive sensor data, including call logs, battery charging, and screen unlocks, were easy to collect and commonly used in other mHealth studies for monitoring sleep, mental health, and depression [7,8,55]. Researchers should be aware of possible exploitation and privacy concerns as we design similar health-related studies. At the same time, there is evidence that these data are already being collected by large companies. Developing individualized interventions directed at the person to increase awareness of vulnerability and potentially developing protective measures may be needed to combat the onslaught of socially engineered content.

### Limitations and Future Work

There are several limitations to the study design that may have affected the performance of passive sensing models. One of these limitations is that the passive sensor data collection was noisy in the sense that user intentions were not fully captured by the current system. For example, it is potentially useful to distinguish screen checks in response to notifications from screen checks initiated by the users. Another limitation is that this study was based on a small sample size, as was the case with previous exploratory passive sensing studies. In addition, due to the cross-platform (iOS and Android) implementation of the mPulse system, the passive sensing and range of mobile sensing modalities were limited. Relevant data sources, such as keyboard and SMS logs, could potentially be used to examine behaviors but were not included in this study because they were only available on the Android platform. Another limitation is that our preference to protect user privacy and reduce battery drain led to the exclusion of relevant mobile sensor data sources, such as location and accelerometer data for motor impulsivity.

Future work should pursue replication of promising measures as well as explore novel sensing modalities with larger samples. Mobile sensor data sources, such as global positioning systems and accelerometers, can be explored to detect mobility and physical activity as predictors of motor impulsivity. Such future work should directly address technical limitations, including battery drain, privacy concerns with regard to location sharing, and the generalizability of mobile sensing models to both iOS and Android platforms. Similarly, physiological sensing modalities from wearable devices, such as heart rate variability, can provide multimodal sensing capabilities. These explorations can reveal more information and improve the prediction accuracy of state impulsivity and impulsive behavior.

### Conclusions

We developed a mobile sensing system called mPulse for both iOS and Android smartphones to remotely detect and monitor state impulsivity and impulsive behavior as part of the DMT study. The design of our mPulse system was based on data that are pervasively available across both iOS and Android platforms: call logs, battery charging, and screen checking. In the exploratory study, we used mobile sensing features to predict trait-based, objective behavioral, and ecological momentary assessment (EMA) of impulsivity and related contacts (ie, risk-taking, attention, and affect).

Our findings suggest that passive sensing features of mobile phones can predict different facets of trait and state impulsivity. For trait impulsivity, the models significantly explained variance in sensation, planning, and lack of perseverance traits but failed to explain motor, urgency, lack of premeditation, and attention traits. On the daily level, the model successfully predicted objective behavioral measures such as present bias in a delay discounting task, commission and omission errors in a cognitive attention task, and total gains in a risk-taking task. Our models also successfully predicted daily EMA questions on positivity, stress, health, and affect. Overall, the study highlights the potential for continuously, passively, and remotely assessing impulsive behavior in daily life to advance the science of self-regulation and awareness.

## Abbreviations

BIS-15: 15-item short form of the Barratt Impulsiveness Scale
DMT: Digital Marshmallow Test
EMA: ecological momentary assessment
GEE: generalized estimating equation
LOSO: leave-one-subject-out
MAE: mean absolute error
mBART: mobile Balloon Analogue Risk Task
mDD: mobile delay discounting task
mGNG: mobile go/no-go task
mHealth: mobile health
PAM: Photographic Affect Meter
PANAS: Positive and Negative Affect Schedule
